# Fluvoxamine and Amantadine: Central Nervous System Acting Drugs Repositioned for COVID-19 as Early Intervention

**DOI:** 10.2174/1570159X19666210729123734

**Published:** 2022-03-28

**Authors:** Konrad Rejdak, Paweł Grieb

**Affiliations:** 1 Department of Neurology, Medical University of Lublin, Lublin, Poland;; 2 Department of Experimental Pharmacology, Mossakowski Medical Research Institute, Polish Academy of Sciences, Warsaw, Poland

**Keywords:** Drug repositioning, SARS-CoV-2, COVID-19, treatment, amantadine, fluvoxamine

## Abstract

***Background*:** As the World faces unprecedented pandemic caused by SARS-CoV-2 virus, repositioning of existing drugs to treatment of COVID-19 disease is urgently awaited, provided that high quality scientific evidence supporting safety and efficacy in this new indication is gathered. Efforts concerning drugs repositioning to COVID-19 were mostly focused on antiviral drugs, or drugs targeting the late phase of the disease.

***Methods*:** Based on published research, the pharmacological activities of fluvoxamine and amantadine, two well-known drugs widely used in clinical practice for psychiatric and neurological diseases, respectively, have been reviewed, with a focus on their potential therapeutic importance in the treatment of COVID-19.

***Results*:** Several preclinical and clinical reports were identified suggesting that these two drugs might exert protective effects in the early phases of COVID-19.

***Conclusion*:** Preclinical and early clinical evidence are presented indicating that these drugs hold promise to prevent COVID-19 progression when administered early during the course of infection.

## 1 INTRODUCTION

In the citation classic published in 2004 Ashburn and Thor [1] defined the term “drug repositioning” and other similar terms (redirecting, repurposing, reprofiling) as the process of finding new uses outside the scope of the original medical indication. Compared to *de novo* discovery and development drug repositioning was characterized by a shorter time span to the registration of a new indication (3-12 *vs*. 10-17 years), reduced safety risk, lower risk of failure, and last but not least by much lower cost. Since then, the situation did not significantly change [2].

Although distinctly faster and less expensive than *de novo* drug development, drug repositioning is a challenging task. Oprea and Overington [3] proposed Drug Repositioning Evidence Level (DREL) scale enabling assessment of the quality of scientific evidence obtained in drug repositioning studies (Table **1**). Although DREL has been designed to assess the quality of studies concerning repurposing of antimicrobials, it has the potential for evaluation of any drug repurposing effort.

The pandemic caused by SARS-CoV-2 virus is particularly predestined for drug repurposing. The number of cases of COVID-19, a disease caused by the pandemic virus, exploded worldwide and within one year exceeded the level of 100 million cases. Obviously, there was no time to develop *de novo* drugs for this disease. Along with unprecedently intense and successful efforts to develop vaccines, multiple preclinical projects and clinical trials have been initiated concerning repositioning various drugs, synthetics as well, as biologicals, to COVID-19. The majority of these endeavors concerned compounds targeting the virus life cycle, or hyperinflammation in the advanced stage of COVID-19. Examples are inhibitors targeting viral RNA-dependent-RNA polymerase previously developed for other RNA viruses (*e.g.* remdesivir originally developed for Ebola, favipiravir developed for pandemic influenza), dexamethasone, monoclonal antibodies hindering immune response (*e.g.* tocilizumab directed toward IL-6 and approved for treatment of rheumatoid arthritis), *etc.* [4-7].

Much less attention has been paid to neurological and psychiatric drugs. Although not mentioned in reviews concerning potential treatments for COVID-19, fluvoxamine and amantadine, known primarily of their activity at the level of the central nervous system, may have potential to become game changers in the fight with COVID-19.

## 2 FLUVOXAMINE

In the Anatomical Therapeutic Chemical Classification System (ATC), fluvoxamine is classified as a drug acting on the nervous system, a psychoanaleptic and antidepressant belonging to Selective Serotonin Reuptake Inhibitors (SSRI) (ATC Code N06AB08). The drug was found to facilitate serotoninergic neurotransmission by selective inhibition of presynaptic reuptake of serotonin in the apparent absence of other major pharmacological effects. Fluvoxamine became one of the first market-approved SSRI antidepressant drugs (initially launched in Switzerland in 1984), and is extensively used since then for treatment of depression and anxiety disorders. Compared to the tricyclic antidepressants, it causes fewer anticholinergic-type and cardiovascular side effects, although the incidence of nausea and vomiting is higher. In patients with depressive illness fluvoxamine offered a suitable alternative, particularly of value for patients with concomitant cardiovascular disease [8].

**Table 1:** Drug Repositioning Evidence Level (DREL) assessment of repositioning studies (based on [3]).

**Drug Repositioning Evidence Level**	**Quality of Scientific Evidence**
0	No evidence; includes in silico predictions without confirmation
1	*In vitro* studies with limited value for predicting *in vivo*/human situation
2	Animal studies with hypothetical relevance in humans
3	Incomplete studies in humans at the appropriate dose *e.g.* proof of concept; few cases from medical records; some clinical effects observed
4	Well-documented clinical end points observed for repositioned drug at doses within safety limits

The premise justifying repurposing fluvoxamine to COVID-19 is related to sigma-1 receptors (S1R), in the context of this drug being a high affinity agonist of these receptors [9]. S1R was discovered as a distinct subtype of opioid receptors in the brain resistant to classical opioid antagonists naloxone and naltrexone [36]. Further research evidenced that these receptors are 223 aminoacid protein residing mainly in the membranes of endoplasmatic reticulum (ER), preferentially at the specific microdomains called mitochondrial-associated membranes. S1R is involved in multiple cellular functions, including chaperoning of proteins passing ER en route to other organelles and regulation of the ER stress response. S1Rs agonists, of which fluvoxamine is the most active (k_i_ = 36 nM in the human brain after single oral administration), increase chaperone activity of S1R protein and alleviate ER stress response [9].

Although S1R is particularly concentrated in brain regions involved in memory, emotion, sensory and motor functions [10], they are ubiquitously present also in a range of peripheral organs [11] and, as recently discovered, are involved in the regulation of inflammation [12]. Considering these findings, Rosen *et al.* [13] explored the potential of S1R activation by fluvoxamine as a means of sepsis control. Their main findings were that fluvoxamine protected mice from lethal septic shock induced with lipopolysaccharide and dampened inflammatory response in human blood leukocytes, suggesting a possible use of the drug to control bacterial sepsis.

Upon infection, SARS-CoV-2 virus hijacks the host cell ER to produce a large quantity of viral glycoproteins, which are set to cause ER stress [14] that inevitably will activate inflammatory signaling and induce inflammatory responses [15]. In COVID-19, lung is among the first organs targeted by viruses. In patients with risk factors that blunt respiratory response to chemical stimuli, lung involvement may lead to blood and tissue hypoxia already in the early stage of the disease [16]. Hypoxia, by itself an ER stress inducing factor [17], will enhance the inflammation and may initiate the cytokine storm. Early pharmacological intervention aimed at alleviating the proinflammatory effect of ER stress may prevent disease progression.

Lenze *et al.* [18] performed a doubly-blind, placebo controlled study to determine whether fluvoxamine given during mild COVID-19 illness prevents clinical deterioration and decreases the severity of the disease. One hundred fifty-two adult COVID-19 patients were randomized to fluvoxamine or placebo treatment starting within 7 days from symptom onset. Over 2 weeks observation clinical deterioration occurred in 0/80 fluvoxamine-treated patients, but it did occur in 6/72 patients treated with placebo. Although the difference was statistically significant (*P*=0.009), the result was considered preliminary. Currently, 3 further trials of fluvoxamine in mild to moderate COVID-19 are registered in the ClinicalTrials.Gov database of clinical studies as recruiting: one phase 3 (NCT04668950 conducted in USA) and two phase 2 studies (NCT04718480 and NCT04711863 conducted in Hungary and South Korea, respectively).

## 3 AMANTADINE

Amantadine has been originally market-approved in USA in 1966 for prophylactic and therapeutic use in influenza type A. Initially, the efficacy in this indication was 90% and the drug gained wide popularity. Its mechanism of action involved interaction with viroporin M2, a component of the viral envelope which, when synthesized by the infected host cell and inserted into the endoplasmic reticulum, facilitates the exit of virions from the cell [19]. In the course of time, quickly mutating influenza A viruses developed resistance, and since 2009 amantadine is not recommended for this indication [20]. In the meantime, further pharmacological properties of amantadine have been discovered. The drug displays pleiotropic activity, and 15 potential repositioning applications have been listed recently [21]. It exerts antiviral effects on some RNA viruses, inhibiting proliferation of Chikungunya virus on Vero cell line [22], and p7 viroporin of hepatitis C virus [23]. It also penetrates blood-brain barrier, acting in the central nervous system as a unique antagonist of glutamate (NMDA) receptors, which concomitantly stimulates glutathione syntesis [24]. Antiinflammatory activity has been noted in the preclinical model of neuroinflammation and sepsis-induced cognitive dysfunction [25]. Amantadine has been successfully repurposed for the treatment of Parkinson's disease [26], and currently it is classified as a drug acting on the nervous system, an anti-parkinson drug, a dopaminergic agent (ATC Code N04BB1). In neurology, it is also prescribed off-label to treat chronic fatigue in multiple sclerosis patients [27], to support neurorehabilitation and correct disorders of consciousness following a traumatic brain injury [28].

In the scientific literature, amantadine in the context of COVID-19 was mentioned few times in March and April 2020. Cimolai [29] postulated that the possible antiviral activity of amnatadine and other similar substances known as adamantanes shall be investigated. Tipton and Wszolek [30] pointed at the antagonism of the drug at the NMDA receptors as potentially beneficial in COVID-19 and suggested that Parkinson’s disease patients taking amantadine could be subject of a retrospective analysis to find out whether the drug could alleviate severe symptoms of this disease. On the basis of *in silico* molecular docking analysis Abreu *et al.* [31] hypothesised that amantadine may block the viroporin channel of COVID-19. Smieszek *et al.* [32] have found that in the *in vitro* screening assay amantadine inhibits the expression of cathepsins L and B, lysosomal proteases involved in the activation of SARS-CoV-2 S protein during virus entry into target cells.

At the end of April 2020, the first observational report on amantadine and COVID-19 has been published describing a group of 15 patients suffering from chronic neurological diseases (Parkinson’s disease or multiple sclerosis) who were infected with SARS-CoV-2 but did not develop symptomatic disease [33]. According to the more recent analysis of the records of patients tested between March and July 2020 for SARS-CoV-2, none of the few patients suffering from Parkinson’s disease and treated with amantadine developed severe complications from COVID-19 [34]. A couple of cases from medical records also seemed to confirm its beneficial effects in COVID-19 [35-36].

There are several possible beneficial effects of amantadine in COVID-19. The hypothesis of blocking SARS-CoV-2 viroporin channel has been confirmed recently in the study of lipid bilayer model systems [37]. The other *in silico* study revealed a possible interaction of amantadine with the amino acids of the receptor-binding domain of SARS-CoV-2 [38]. A direct antiviral effect should not be excluded or neglected, but other possibilities are also worth consideration. Recently, it has been proposed that possible benefits of amantadine in COVID-19 may be related to its anti-fatigue and arousal-enhancing effects, which may correct blunted sensitivity of the respiratory system to chemical stimuli, in particular to hypoxia [39]. Counteracting the development of hypoxia early in the course of COVID-19 may have a crucial role in the prevention of the disease evolution toward serious and fatal outcome [16]. Amantadine is also an agonist of sigma-1 receptors. Although this interaction (with k_i_ estimated as 7.4 - 14 µM) is much weaker than that of fluvoxamine, at concentration range covered by ususal therapeutic doses amantadine is expected to significantly bind to S1R [40].

Currently, a placebo-controlled trial of amantadine for mild-to-moderate COVID-19 in high-risk patients is being set up in Poland (EudraCT 2021-001144-98). Other adamantanes like memantine remain of interest with similar indication, but currently, there is no sufficient evidence to support it [33].

## 4 THE CASE FOR EARLY TREATMENT OF COVID-19

The spectrum of COVID-19 disease caused by SARS-CoV-2 virus infection ranges from asymptomatic infection to severe pneumonia with acute respiratory distress syndrome (ARDS) and death. In the majority of cases, the illness is self-limited without any specific treatment, but up to 20% of infected patients evolve toward a severe stage with high mortality. Several risk factors of dangerous evolution of the disease have been identified, including old age, obesity, diabetes, sleep apnea and other conditions characterized by the blunted response of respiration to hypoxia (see [16] and the references cited).

According to NIH guidelines (files.covid19 treatmentguidelines.nih.gov/guidelines/section/section_43.pdf), currently no drug has been shown to prevent the development of infection when given before or after exposure to SARS-CoV-2, i.e. as preexposure or postexposure prophylaxis. No specific therapy is currently recommended for mild-to-moderate COVID-19 patients, and only recently an urgent need of treatments to be administered early during the course of infection to prevent disease progression and its long-term complications has been recognized [41]. Although intravenous monoclonals targeting spike protein of SARS-CoV-2, bamlanivimab (LY-CoV555, Lilly) and casirivimab+ imdevimab cocktail (Regeneron), received Emergency Use Authorizations issued by FDA for use in outpatient settings, these drugs will be expensive (projected price 1,250-1,500 USD per dose) and in short supply, and their use will remain restricted [42].

## CONCLUSION

As remarked by Ayres [43], in the fight with COVID-19, scientists wrongly focused on developing antivirals instead of drugs that promote physiological function during the infection. Fluvoxamine and amantadine given early in the course of this disease may, in a sense, act in such a way. Both are inexpensive and well tolerated generics. However, because at the moment neither drug deserves DREL score higher than 2 or 3, further clinical studies of their safety and efficacy in mild-to-moderate COVID-19 are undoubtedly required.
